# Predictive Accuracy of C-reactive Protein as an Early Indicator of Leakage After Anastomosis and Primary Repair in Gastrointestinal Surgery

**DOI:** 10.7759/cureus.71107

**Published:** 2024-10-08

**Authors:** Shaswat Mayank, Nishith M Ekka, Dipendra K Sinha, Binay Kumar, Nabu Kumar, Abhinav Ranjan, Somya Verma, Srishti Darad, Sourabh Kumar, Niharika Mayank

**Affiliations:** 1 General Surgery, Rajendra Institute of Medical Sciences, Ranchi, IND; 2 Surgery, Rajendra Institute of Medical Sciences, Ranchi, IND; 3 General Medicine, Institute of Medical Sciences, Banaras Hindu University, Varanasi, IND

**Keywords:** anastomotic leak after gastrointestinal surgery, c-reactive protein (crp), gastrointestinal perforation, postoperative mortality, prognostic role

## Abstract

Background

Anastomotic leakage after gastrointestinal surgery is a significant postoperative complication that leads to increased morbidity and mortality. C-reactive protein (CRP) has been used previously as a predictive marker of anastomotic leakage in gastrointestinal surgeries. Its short half-life makes it a reliable marker for postoperative complications, rapidly returning to normal values as the patient recovers. We conducted this study to evaluate the predictive accuracy of serum CRP levels in predicting anastomotic leaks in patients undergoing gastrointestinal repair surgeries.

Methods

Ninety-six gastrointestinal surgeries involving anastomoses and primary repairs were included in the study. CRP was taken serially from postoperative days (POD) 1 to 7. Patients were divided into two groups based on postoperative outcomes: leakage and non-leakage. The receiver operating characteristic (ROC) curve of CRP levels with leak and mortality was plotted to find a threshold value for leak and mortality.

Results

Out of 96 patients included in the study, Group B (non-leakage) consisted of 78 patients (81.3%), while Group A (leakage) comprised 18 patients (18.7%). ROC analysis identified a CRP cutoff of 127 mg/L on POD 5, with high sensitivity (80%) and high specificity (80%) indicating a high likelihood of leakage above this threshold.

Conclusion

This study underscores the importance of monitoring CRP levels in the postoperative period, particularly on POD 5, as a non-invasive and cost-effective tool for the early detection of anastomotic leaks.

## Introduction

Anastomotic leakage after gastrointestinal surgery is a significant postoperative complication that leads to increased morbidity and mortality [1-3]. It occurs when a patient, having undergone gastrointestinal repair surgery, exhibits fecal or bilious content in a drain, signs of peritonitis, or air or fluid collection near the surgical site as seen in a CT scan. The rate of anastomotic failure varies depending on the site of the anastomosis. In colorectal surgery, the incidence of leaks ranges from 2% to 20%, with mortality rates reaching as high as 29.4% [2,4].

Routine imaging to diagnose leaks is neither cost-effective nor entirely reliable and carries the drawback of radiation exposure. Therefore, a cost-efficient and sensitive serum marker would be highly beneficial for the safe discharge of patients. C-reactive protein (CRP) has been used to diagnose surgical infections within the abdomen [4-8]. CRP is an acute-phase protein produced in response to acute conditions, primarily stimulated by IL-6 during the acute phase of inflammation or infection [9]. A CRP level exceeding 150 mg/dL necessitates further examination for leaks, particularly if clinical findings are concerning [10]. While CRP has been extensively studied as a predictor of anastomotic leakage in colorectal cancer surgeries [10-11], its significance in gastrointestinal anastomotic leaks, in general, has not been widely evaluated. This study aims to assess the predictive accuracy of serum CRP levels in diagnosing anastomotic leaks in patients undergoing gastrointestinal repair surgeries.

## Materials and methods

**Figure 1:**
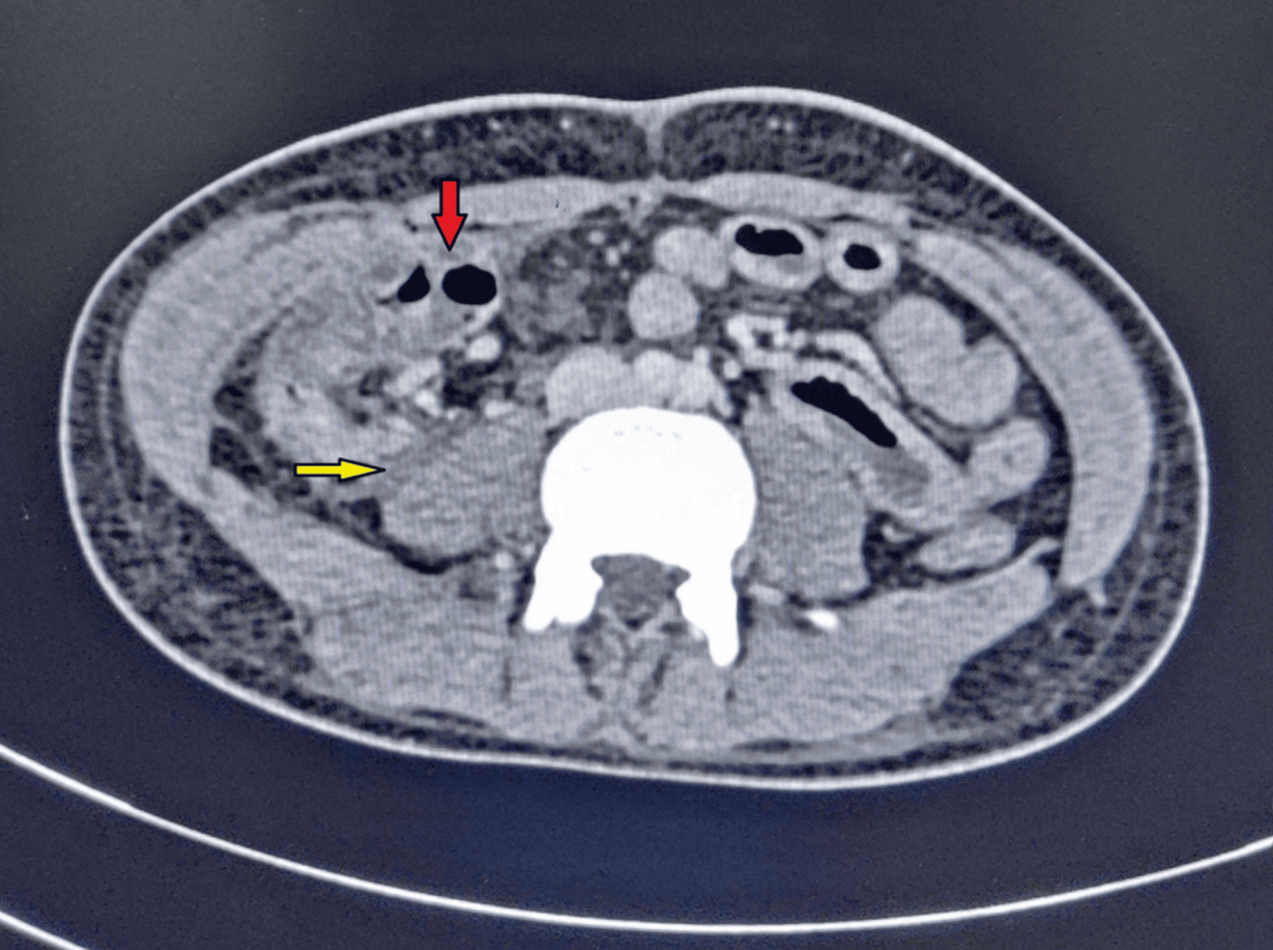
CECT of the whole abdomen showing anastomotic leak (yellow arrow) after ileo-ileal anastomosis (red arrow) CECT: contrast-enhanced computed tomography

**Figure 2:**
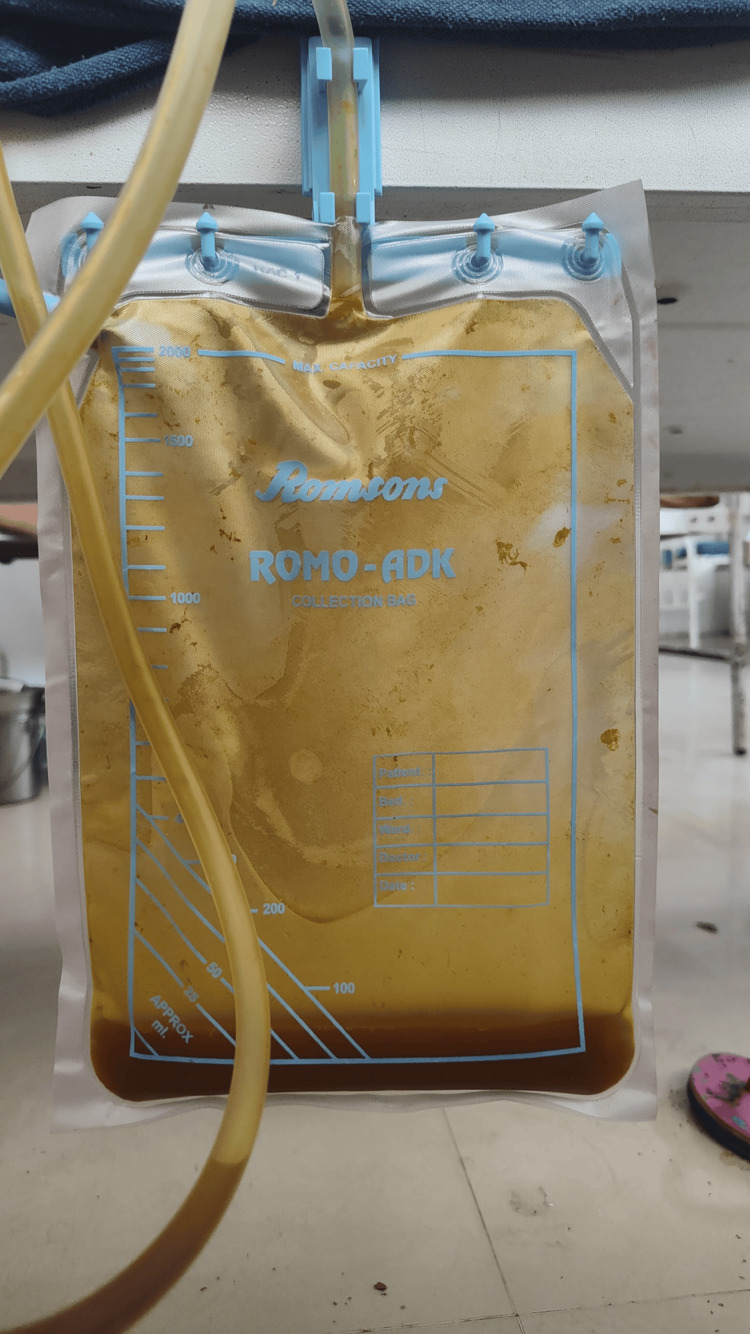
Abdominal drain illustrating anastomotic leak

Ninety-six gastrointestinal surgeries, two elective, and 94 emergency cases involving anastomoses and primary repairs were performed in the General Surgery Department of Rajendra Institute of Medical Sciences, Ranchi, from November 2022 to April 2024. However, multiple surgeons were included in the study; only the single-layer extramucosal hand-sewn technique for anastomosis and primary repair done by interrupted extramucosal suturing were included. The clinical and radiological criteria used for defining leak were (1) the presence of air or fluid near the site of anastomosis identified on CT (Figure 1), (2) feculent or bilious secretion through the drain, and (3) clinical signs of peritonitis and/or presence of fecal or purulent discharge during surgical re-approach (Figure 2). This study was self-financed. The Institutional Ethics Committee of Rajendra Institute of Medical Sciences approved this study (approval number: 238/26.10.2023).

Inclusion and exclusion criteria

Inclusion criteria include patients of both genders, between the age group of 18 and 60 years, and patients who underwent gastrointestinal repair surgery in the Department of General Surgery. Exclusion criteria include patients with chronic organ failure, known malignancy, immunocompromisation, and patients who underwent diversion ileostomy.

Sample size

The sample size was 92, which was calculated based on a prevalence of 9.6%, a confidence level of 95, and a margin of error of 6. Prevalence was taken from Singh et al. [10].

Grouping and data collection

Patients were divided into two groups based on postoperative outcomes: leakage (n=18) and non-leakage (n=78). Since the patients were prospectively enrolled at the time of surgery and were divided into groups based on outcome, there is an unequal division of patients into groups. The clinical and demographic characteristics of these groups are shown in Table 1.

**Table 1:** Demographic and clinical characteristics Quantitative variables were defined by mean and SD and compared using Student’s t-test. Qualitative variables were determined by the number of cases and percentages and compared using the chi-square test. A p-value <0.05 was considered statistically significant. The Pearson Chi-square test was used for comparison, and if group counts were <5, Fisher's exact test was applied. P-values were obtained using the Mann–Whitney U test. SD: standard deviation

Characteristics	Total (n=96) (%)	Non-leakage (n=78) (%)	Leakage (n=18) (%)	p-value	x²	Fisher's exact test	T-value	
							Right tail	Two-tailed
Age, median (range)	39 (18-60)	78 (78.8)	18 (18.2)	0.866	50.6	0.023	1.68	+/-2.02
Gender, n (%)	-	-	-	0.766	0.0912	1	-1.085	+/- 0.39
Female	24 (25)	20 (25.64)	4 (22.2)	-	-	-	-	-
Male	72 (75)	58 (74.35)	14 (77.7)	-	-	-	-	-
Surgical indication, n (%)	-	-	-	0.478	3.1	0.784	-0.43	+/- 0.475
Annular pancreas	1 (1)	0	0	-	-	-	-	-
D1 perforation	57 (59.4)	44 (56.4)	13 (72.22)	-	-	-	-	-
Gastric antrum perforation	22 (22.9)	20 (25.64)	2 (11.11)	-	-	-	-	-
Ileosigmoid knotting	1 (1)	1 (1.28)	0	-	-	-	-	-
Jejunal perforation	15 (15.6)	3 (3.84)	3 (16.66)	-	-	-	-	-
Surgical procedure, n (%)	-	-	-	0.958	1.19	0.895	-1.38	+/- 0.16
Duodenojejunostomy	1 (1)	1 (1.2)	0	-	-	-	-	-
Graham's patch repair	2 (2)	2 (2.56)	0	-	-	-	-	-
Jejunoileal and rectosigmoid anastomosis	1 (1)	1 (1.2)	0	-	-	-	-	-
MGP repair	73 (76)	58 (74.35)	15 (83.33)	-	-	-	-	-
Primary repair	19 (19.8)	16 (20.51)	3 (16.77)	-	-	-	-	-
Death, n (%)	-	-	-	<0.001	22.9		6.31	+/- 12.7
No	91 (94.8)	78 (100)	13 (72.22)	-	-	-	-	-
Yes	5 (5.2)	0	5 (27.7)	-	-	-	-	-

CRP measurement

Serum CRP levels were measured serially from postoperative day (POD) 1 to 7 using immunoassays with the turbidimetric method on an Architect Plus C4000 analyzer (Abbott Laboratories, Chicago, IL, USA). CRP levels greater than 5 mg/L were considered altered [12].

Postoperative monitoring

Daily monitoring of the patients was done, including temperature, bowel movement, abdominal pain, and the content of the abdominal drain. To confirm the presence of anastomotic leakage, patients with altered parameters underwent additional laboratory and imaging tests, such as radiography or CT scans.

Prophylactic measures

All patients received antibiotic prophylaxis. The use of mechanical colon preparation was restricted to patients undergoing elective treatments. This comprehensive methodology ensured systematic data collection and reliable CRP measurements, facilitating the analysis of CRP levels as a predictive marker for anastomotic leaks in gastrointestinal surgeries.

Statistical analysis

Statistical analyses were performed using SPSS Statistics version 29.0 (IBM Corp. Released 2023. IBM SPSS Statistics for Windows, Version 29.0.2.0 Armonk, NY: IBM Corp.) and Jamovi version 2.5 (retrieved from https://www.jamovi.org). Quantitative variables were defined by mean and standard deviation (SD). They were compared using the Student's t-test. Qualitative variables were defined by the number of cases and percentages. The Pearson Chi-square test was used for comparison, and if group counts were <5, Fisher's exact test was applied [13]. A p-value <0.05 was considered statistically significant.

To assess the predictive value for the diagnosis of anastomotic leak, a receiver operating characteristic (ROC) curve analysis was conducted. These parameters' negative predictive value (NPV), positive predictive value (PPV), sensitivity, and specificity were calculated.

## Results

A total of 96 patients were included in the study, comprising 24 females (25%) and 72 males (75%), yielding a male-to-female ratio of 3:1. The mean age of the patients was 39 ± 12.9 years. Patients were categorized into the following age groups: 32 patients (33.3%) were between 18 and 30 years, 18 patients (18.8%) were between 31 and 40 years, 25 patients (25%) were between 41 and 50 years, and 21 patients (21.9%) were between 51 and 60 years (Table 1).

Group B (non-leakage) consisted of 78 patients (81.3%), while Group A (leakage) comprised 18 patients (18.7%). Most patients in the leakage group were between 18 and 30 years old. In Group A, five patients (5%) did not survive, with most fatalities occurring in the 18-30 age group. Duodenal perforation was diagnosed in 57 patients (59.4%). Among those who developed leaks, 83.33% underwent modified Graham’s patch repair (Table 1).

**Figure 3:**
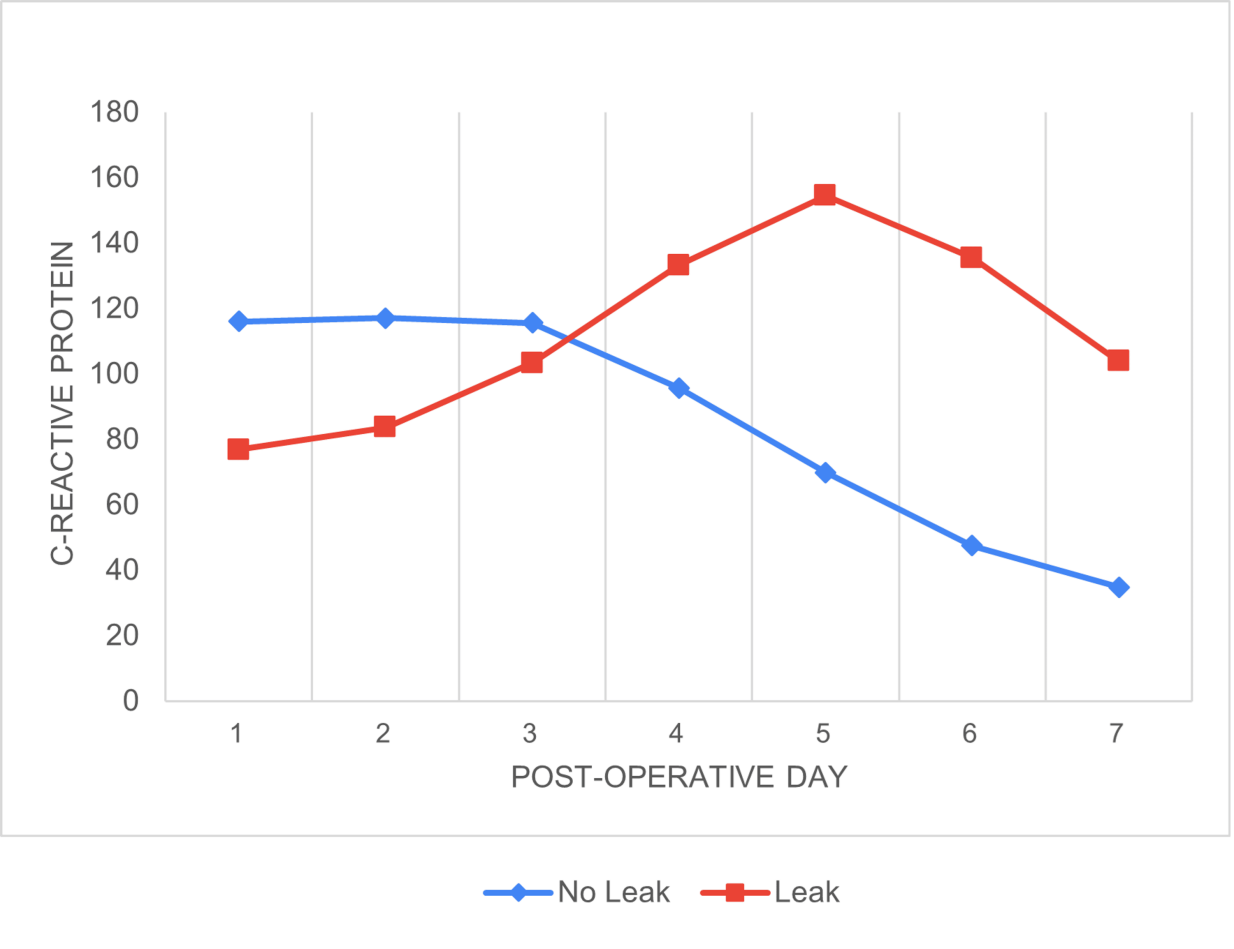
CRP levels in patients with and without leak from POD 1 to 7 From POD 1 to 5, there was an upward trend in the mean CRP levels of leak patients. In contrast, CRP levels in non-leak patients declined from POD 3. CRP: C-reactive protein, POD: postoperative day

**Figure 4:**
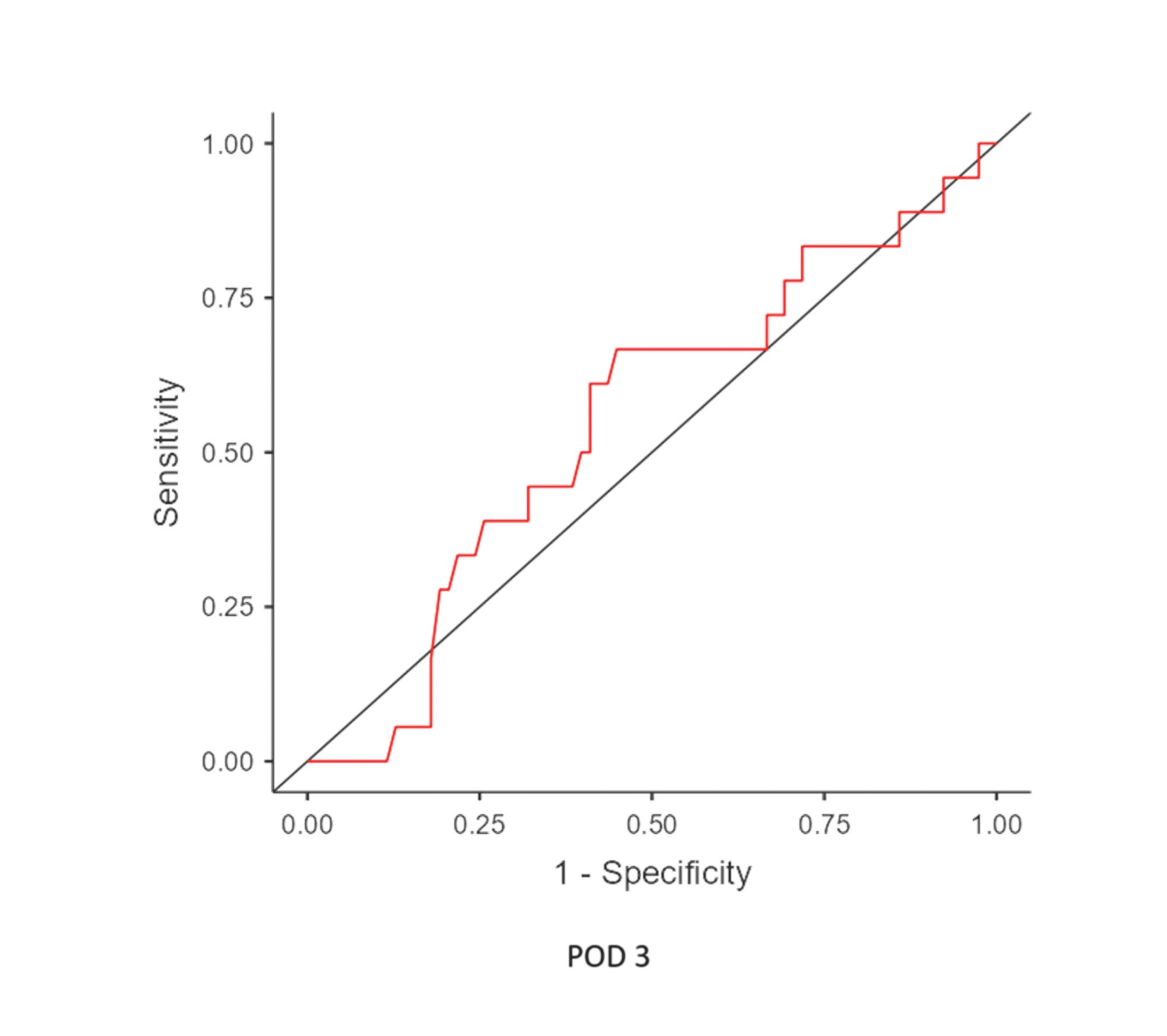
ROC curve of CRP level from POD 3 ROC: receiver operating characteristic, CRP: C-reactive protein, POD: postoperative day

**Figure 5:**
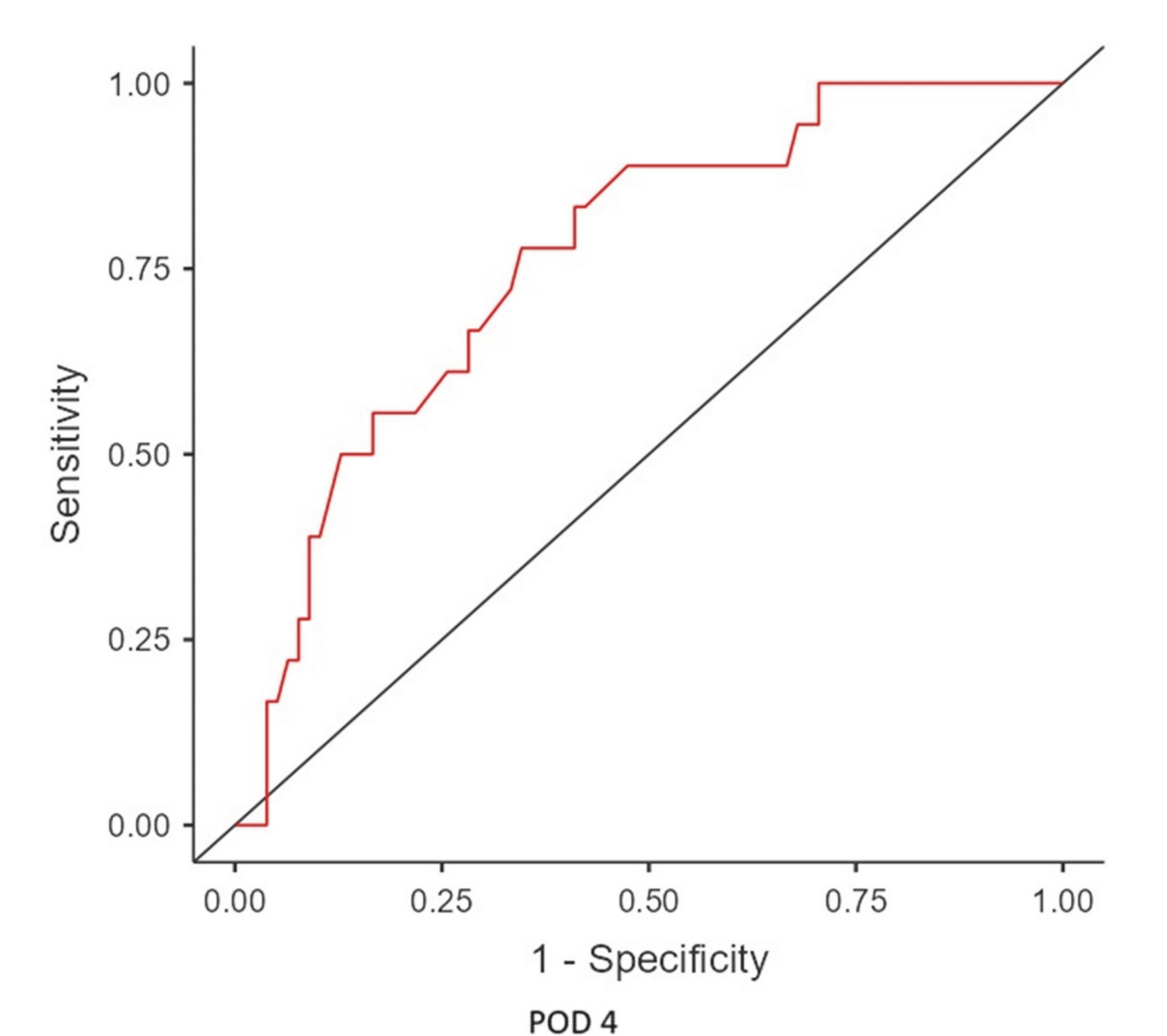
ROC curve of CRP level from POD 4 ROC: receiver operating characteristic, POD: postoperative day, CRP: C-reactive protein

**Figure 6:**
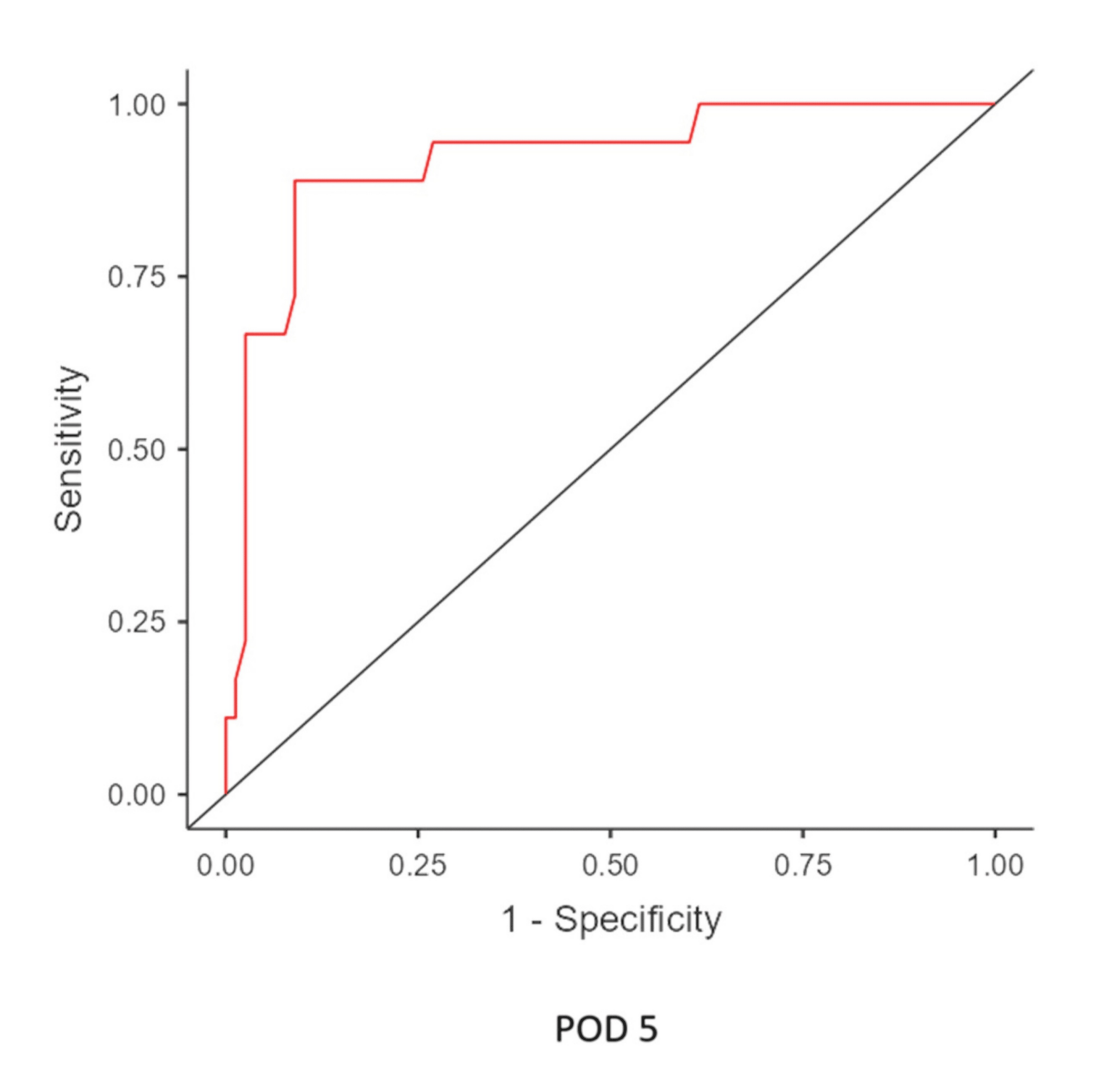
ROC curve of CRP level from POD 5 ROC: receiver operating characteristic, CRP: C-reactive protein, POD: postoperative day

The mean time of leak was postoperative day 7.12, and the median leak time was POD 7. From POD 1 to 5, there was an upward trend in the mean CRP levels of leak patients. In contrast, CRP levels in non-leak patients declined from POD 3. (There is an initial rise immediately 24-48 hours postoperatively [4]) (Figure 3). ROC analysis identified a CRP cutoff of 127 mg/L on POD 5, with a sensitivity of 80%, specificity of 80%, NPV of 98.65%, and PPV of 18.18%, indicating a high likelihood of leakage above this threshold (Figures 4-6) (Table 2).

**Table 2:** Results of ROC analysis ROC curve analysis on POD 3 (cutoff value, 119 mg/L), POD 4 (cutoff value, 99 mg/L), and POD 5 (cutoff value, 127 mg/L) ROC: receiver operating characteristic, POD: postoperative day, NPV: negative predictive value, PPV: positive predictive value, AUC: area under the curve

POD	Accuracy	Sensitivity	Specificity	NPV	PPV	AUC
POD 3	0.813	0.60	0.681	0.97	0.093	0.619
POD 4	0.802	1.0	0.54	1.0	0.1	0.774
POD 5	0.913	0.8	0.802	0.987	0.18	0.859

## Discussion

Anastomotic leakage is a significant and feared complication in the postoperative period, with incidence rates varying across different types of surgeries [14]. Maghsoudi and Ghaffari reported a leak rate of 4% following Graham’s patch repair, with a notably high mortality rate of 29.4% [15]. The early and accurate prediction of anastomotic leaks remains a clinical challenge. Routine imaging techniques, while informative, are not always cost-effective or reliable and involve radiation exposure. Hence, there is a critical need for a reliable predictive tool to facilitate early leak detection and thereby reduce morbidity and mortality.

Various biomarkers, including procalcitonin, CRP, IL-6, and TNF-α, have been explored for their potential in early leak detection. CRP stands out due to its cost-effectiveness, minimally invasive nature, availability, and predictive value [16-17]. Alves et al. reported an 18% mortality rate associated with the delayed diagnosis of anastomotic leaks (POD 5), whereas morbidity was minimal when leaks were detected and treated before POD 5 [16]. McDermott et al.'s meta-analysis identified a CRP level exceeding 150 mg/L on PODs 3-5 as the most sensitive marker for leaks post-colorectal surgery [17]. Consistent with these findings, our study identified a CRP cutoff of 127 mg/L on POD 5, which demonstrated high sensitivity, specificity, and NPV for predicting anastomotic leaks.

Su’a et al. highlighted significant variations in CRP cutoff values for predicting anastomotic leaks [18], which are influenced by the surgical approach and the specific day of CRP measurement. Waterland et al. observed higher CRP levels in open surgeries than in laparoscopic surgeries, with a cutoff of 123.5 mg/L on POD 4 predictive of leaks in conventional surgeries [19]. Despite these variations, multiple studies support the utility of CRP in early leak detection.

Our study is unique in evaluating CRP's predictive role in gastrointestinal repair surgeries beyond colorectal surgeries. Most of our patients had duodenal perforations, followed by gastric perforations and small bowel anastomoses. Although our study's small sample size and single-center design are limitations, the findings suggest that a CRP level of 127 mg/L on POD 5 is a reliable predictor of anastomotic leaks.

## Conclusions

This study underscores the importance of monitoring CRP levels in the postoperative period, particularly on POD 5, as a non-invasive and cost-effective tool for the early detection of anastomotic leaks. We recommend using a CRP cutoff level of 127 mg/dL on POD 5 in patients who underwent gastrointestinal repair surgery. This can improve patient outcomes by enabling timely interventions and reducing the associated morbidity and mortality. Limitations include the study being single-center with a comparatively small sample size, which requires a multicentric study for validation. Further study may be required to validate our findings.
